# Fabrication of Piezoelectric Polymer and Metal–Organic Framework Composite Thin Films Using Solution Shearing

**DOI:** 10.1021/acsami.5c07907

**Published:** 2025-07-23

**Authors:** Ankit Dhakal, Sangeun Jung, Byungjoon Bae, Ajith Mohan Arjun, Sean Robinson, Yongmin Baek, William T. Riffe, Emma M. Tiernan, Shubha Gunaga, Prince Verma, Meagan R. Phister, Madison Stone, Kevin H. Stone, Amanda Morris, Nathan S. Swami, Patrick E. Hopkins, Amrit Venkatesh, Kyusang Lee, Gaurav Giri

**Affiliations:** † Department of Chemical Engineering, 2358 University of Virginia, Charlottesville, Virginia 22904-4746, United States; ‡ Department of Electrical and Computer Engineering, University of Virginia, Charlottesville, Virginia 22904-4746, United States; § Department of Materials Science and Engineering, University of Virginia, Charlottesville, Virginia 22904-4746, United States; ∥ Department of Mechanical and Aerospace Engineering, University of Virginia, Charlottesville, Virginia 22904-4746, United States; ⊥ Department of Physics, University of Virginia, Charlottesville, Virginia 22904-4746, United States; # Stanford Synchrotron Radiation Lightsource, SLAC National Accelerator Laboratory, Menlo Park, California 94025, United States; ¶ Department of Chemistry, Virginia Tech, Blacksburg, Virginia 24061, United States; ∇ 189689 National High Magnetic Field Laboratory, Florida State University, Tallahassee, Florida 32310, United States

**Keywords:** metal−organic framework, piezoelectricity, UiO-66, P(VDF-TrFE)

## Abstract

Polymer-metal–organic framework (polymer-MOF) composites have garnered significant interest as polymers can enhance the processability and industrial applicability of MOFs. Thin films of these composites are particularly attractive for applications in sensing, separations, and flexible electronics. Solution shearing, a meniscus-guided coating technique, has emerged as a scalable process for fabricating thin films of MOFs, and can produce large-area films within minutes. In this study, we utilized solution shearing to fabricate composite thin films of a MOF UiO-66 and a piezoelectric polymer poly­(vinylidene fluoride-trifluoroethylene) (P­(VDF-TrFE)), investigating how polymer concentration during MOF synthesis and composite formation influences thin film properties, including crystallinity, surface coverage, and piezoelectric performance. Additionally, solid-state NMR spectroscopy was utilized to probe the interactions between P­(VDF-TrFE) and UiO-66 in the composite. Evidence from solid-state NMR indicated polymer-MOF interactions, suggesting that the polymer strands are in close proximity to the UiO-66 pores, supporting a mixed surface coating and pore infiltration model. Furthermore, incorporating P­(VDF-TrFE) enhanced the film’s areal coverage from 70% to 100%. While the thermal conductivity remained essentially unchanged, the composite film showed an improved piezoelectric effect. The composite with 91 wt % P­(VDF-TrFE) exhibited the highest output voltage of 9.1 V and a sensitivity of 0.26 V/N under applied pressure. This work demonstrates the potential of solution shearing as a scalable technique for fabricating polymer-MOF composite thin films.

## Graphical abstract

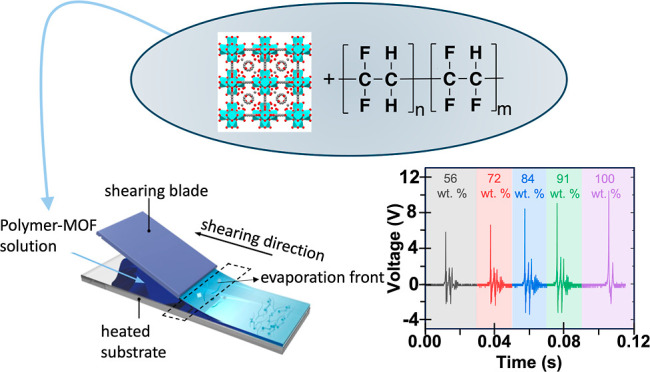

## Introduction

Metal–organic frameworks (MOFs), a class of hybrid porous crystalline materials composed of inorganic nodes and organic linkers, have garnered significant attention in recent years due to their structural and chemical diversity, tunable porosity, and large specific surface area. The vast selection of metal ions/clusters and organic linkers enables the synthesis of MOFs with diverse properties, including tunable surface areas, pore sizes, and functionalities, making them highly adaptable for a wide range of applications,^1^ such as sensing,^2^ adsorption,^3^ separation,^4^ drug delivery,^5^ and catalysis.^6^

The stability of MOFs toward temperature, solvents, and humidity is crucial for their practical applications. However, many MOFs are highly sensitive to moisture, high temperature, and environmental pH, limiting their commercial utilization.^7^ Zirconium (Zr)-based MOFs, on the other hand, are known for their exceptional chemical, thermal, and mechanical stability, making them desirable for the aforementioned applications.^8,9^ One drawback is that many Zr-MOFs possess intrinsic brittleness and a powder-like form when synthesized. These inherent properties present significant challenges in processability and large-scale industrial applicability for this class of MOFs.^10^

Polymer incorporation with MOFs to form polymer-MOF composites to enhance processability has been an expanding area of research in recent years.^11,12^ Adding polymers to MOFs introduces the polymers’ intrinsic flexibility and processability into the composite. Moreover, polymers may impart unique properties, such as electron conductivity,^13^ piezoelectricity,^14^ specific molecular adsorption,^15^ etc., to the composite. Gandara-Loe et al. demonstrated that incorporating UiO-67 into the polyurethane (PU) matrix enhanced the loading of the drug brimonidine tartrate and resulted in prolonged release, compared to the polymer control.^16^ Melvin et al. showed that the composite of H_3_[(Cu_4_Cl)_3_(BTTri)_8_] (CuBTTri) MOF and PU showed better catalytic properties in comparison to the MOF powder alone in converting S-nitrosoglutathione (GSNO) to nitric oxide (NO).^17^ By combining the high surface area and tunable porosity of MOFs with the flexibility, processability, and mechanical strength of polymers, polymer-MOF composites offer a synergistic integration to enhance the overall practicality and applicability of MOFs.

Different strategies have been applied to form polymer-MOF composites.^18^ One common technique is to disperse monomers into the pores of the MOF, followed by polymerization to create the polymer-MOF composite.^18^ Shanahan et al. synthesized PANI (poly aniline) @UiO-66 by initiating polymerization of aniline in the pores of UiO-66 for application as tunable semiconducting materials.^13^ However, controlling the polymerization reaction can be challenging and lead to inhomogeneous polymerization in the composite. Another method of making polymer-MOF composites involves introducing preformed polymers to a suspension of preformed MOFs.^19^ Duan et al. have utilized this technique to synthesize composite membranes of poly­(vinylidene fluoride) (PVDF) and poly­(ethylene oxide) (PEO) with UiO-66 to study polymer infiltration into MOFs using solid-state nuclear magnetic resonance (NMR) spectroscopy.^20^ With this synthesis technique, the entrapment of the polymer is entirely dependent on the diffusion of the polymer chains through the MOF pore windows.^20^ This diffusional constraint might lead to blockage of MOF pores, limits the use of polymers with large, branched backbones, and reduces the overall polymer loading.

To overcome inhomogeneous polymer distribution and pore plugging, the MOFs can be crystallized around preformed polymers, i.e., in situ MOF synthesis.^19,21^ Li et al. synthesized PEDOT: PSS@HKUST-1 by first mixing positively charged copper hydroxide nanostrands (CHNs) with negatively charged PEDOT: PSS, then subsequently adding the mixture to the linker solution, leading to the synthesis of HKUST-1 around the polymer structure.^22^ Since the polymer loading is not limited by the diffusion of the polymer chains into the MOF pores, higher loading of polymers can be achieved, and the reaction time scale is solely dependent on the rate of MOF formation and growth around the polymer chains.

Given the expanding range of applications, significant attention has also been directed toward fabricating polymer-MOF composites as thin films for use in sensors,^23^ electronics,^24^ electrocatalysis,^25^ etc. Techniques such as layer-by-layer growth,^26^ solvothermal growth,^27^ spin coating,^21^ and drop casting^28^ have been previously used to deposit polymer-MOF composite thin films. However, these techniques are challenging to scale up for industrial applications. Recently, a meniscus-guided coating technique, known as solution shearing, has emerged as an effective method for rapidly fabricating large-area thin films of MOFs and polymers.^29–32^ In this technique, a precursor solution is sandwiched between a moving blade and a heated substrate. As the blade moves, a meniscus is formed between the blade and the substrate. The solvent evaporates through this meniscus, resulting in the subsequent formation of a solid film. Large-area thin films can be easily created using solution shearing since the evaporation front is independent of the substrate width.^30,31^ Thin film properties such as thickness, crystallinity, and coverage can be regulated using different shearing parameters (temperature, blade speed, concentration, and solvent).^33,34^ Recently, Verma et al. used solution shearing to prepare thin films of Zr-based MOFs (UiO-66, NU-901, and MOF-525) and utilized MOF-525 (Fe) for electrocatalytic conversion of CO_2_ to CO.^30,31^ Despite multiple studies focusing on the use of solution shearing for either polymer film fabrication or MOF film fabrication, to the best of our knowledge no study has yet demonstrated the use of solution shearing to fabricate polymer-MOF composite thin films.^29–31,35^

In this study, we fabricated thin films of poly­(vinylidene fluoride-trifluoroethylene) P­(VDF-TrFE)-UiO-66 polymer-MOF composite using the solution shearing technique. UiO-66 is a Zr-based MOF consisting of Zr-oxo clusters as nodes and terephthalic acid as the linker (Figure 1a).^36^ P­(VDF-TrFE) is a well-studied piezoelectric polymer used widely in sensors, generators, and transducers.^37^ The piezoelectric behavior of P­(VDF-TrFE) arises specifically from its crystalline β-phase, where the polymer chains adopt an all-trans conformation (Figure 1b).^38^ Here, we studied the effects of modifying the composite’s relative concentration of MOF and polymer on the resulting film properties, including crystallinity, piezoelectricity, and conductivity. We also probed the interactions between the polymer and the MOF in the composite by utilizing solid-state NMR spectroscopy. Our findings indicate that increasing the polymer concentration enhances the piezoelectric performance of the composites, while the thermal conductivities remain relatively unchanged. Notably, the composite thin films demonstrated a high output voltage of 9.1 V and excellent sensitivity (0.26 V/N) when subjected to pressure, comparable to the other MOF-PVDF-based films reported in the literature.^24,39^ Furthermore, we demonstrate solution shearing as a scalable technique for fabricating polymer-MOF composite films. These findings suggest the potential usefulness of the solution-sheared composite films for flexible electronics and sensing applications.

**Figure 1:**
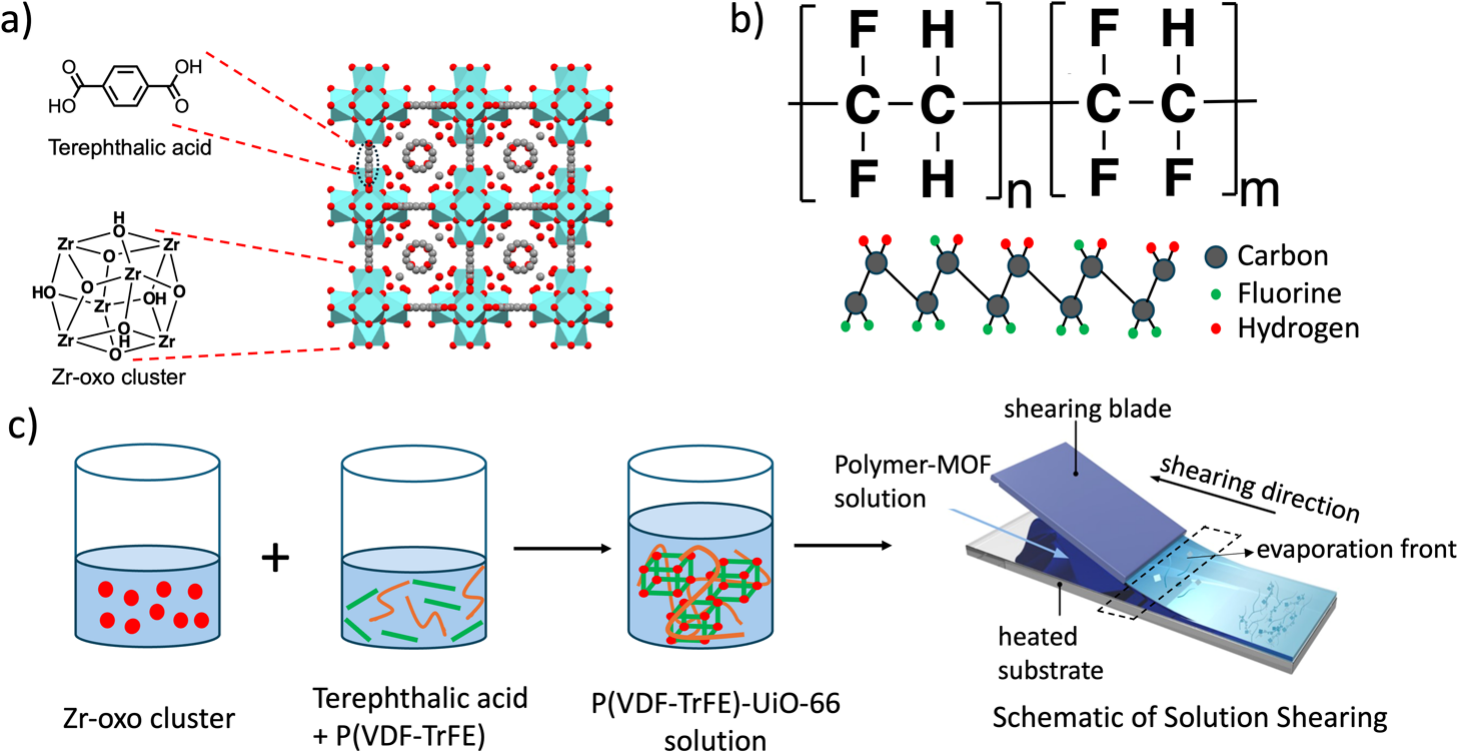
(a) Crystal structure of UiO-66.^40^ (b) Molecular structure of P­(VDF-TrFE) polymer, and the schematic for β-phase. (c) Process of P­(VDF-TrFE)-UiO-66 thin film synthesis using in situ MOF crystallization, followed by solution shearing (red dots represent the node, green lines represent the linker, and the orange lines represent the polymer).

## Materials and Methods

### Materials

Dimethylformamide (DMF, 99.8%), toluene (99.9%), acetone (99.9%), methanol (≥99.9%), acetic acid, zirconium­(IV) propoxide (70% in propanol), benzene-1,4-dicarboxylic acid (H_2_BDC, 98%), polyvinylidene fluoride-trifluoro ethylene (PVDF-TrFE) (solvene 250/P400), trichloro­(octadecyl)­silane (OTS, ≥ 90%) were purchased from Sigma-Aldrich and used as received. Indium tin oxide (ITO) coated glass slides (1.1 mm thick and 100 Ω/sq), glass slides (1 mm thick), and isopropyl alcohol (IPA) were purchased from Fisher Scientific. Silicon wafer (285 nm thick silicon dioxide layer) was purchased from University Wafer.

### Substrate and Shearing Blade Preparation

Glass and ITO-coated glass slides were cut into 1-in. × 0.5-in. rectangular pieces and used as substrates. They were washed by sonicating in methanol for 10 min and then dried using an air gun. A silicon wafer was used to prepare the shearing blade. First, a circular silicon wafer was cut into an appropriate size such that the flat side could be used as the shearing blade. Then, it was sonicated in IPA for 10 min and dried using an air gun. The silicon wafer was then placed in UV/ozone for 20 min. In a crystallization dish, 200 μL of OTS was mixed with 100 mL of toluene. The UV/ozone silicon wafer was placed in the OTS-toluene solution and stirred overnight at 50 °C. For additional chemisorption to occur after removal from the solution, the wafer was dried and annealed at 90 °C for 1 h. The wafer was sonicated in acetone for 5 min to remove any physisorbed OTS.

### Synthesis of P­(VDF-TrFE)-UiO-66 Composite Solution

The synthesis procedure was adapted from Farha et al.^41^ To synthesize the Zr-oxo cluster node solution, 355 μL of 70% zirconium propoxide solution in 1-propanol (0.79 mmol) was added to a mixture of 4 mL acetic acid and 7 mL DMF in a 20 mL vial. The solution was then sonicated for 10 min and placed in an oven at 130 °C for 2 h, after which the solution changed from colorless to yellow. In another vial, different weight percentages of P­(VDF-TrFE) (Table S1) were added to 5 mL of DMF and stirred at 60 °C for 2 h. Then, 115 mg of terephthalic acid was added to the polymer solution and stirred until fully dissolved. Then, 5 mL of the node solution was added to the linker and polymer solution and mixed overnight. The synthesized polymer-MOF solution was then used to fabricate thin films via solution shearing.

### Solution Shearing of P­(VDF-TrFE)-UiO-66 Composite Solution

First, the shearing blade was rinsed with toluene, acetone, and IPA, respectively, and dried with the air gun. The stage was then heated to 90 °C, the shearing blade was held in place using a vacuum at the top, and the cleaned substrate was held in place using a vacuum at the bottom stage. Then, 40–70 μL of the polymer-MOF composite solution was added between the blade and the substrate, and the blade was moved at a speed of 0.05 mm/s. The solvent evaporated through the meniscus between the blade and the substrate, leading to the fabrication of the composite film. As annealing P­(VDF-TrFE) films between their Curie temperature and melting temperature has been shown to enhance their piezoelectric performance, the fabricated composite films were annealed for 2 h at 130 °C.^42^ The films were then stored for further characterization.

### Synthesis of Physical and In Situ Composite Mixtures of P­(VDF-TrFE) and UiO-66 for Solid-State NMR Spectroscopy

#### Physical Mixture

To synthesize UiO-66, 5 mL of node solution was mixed with 5 mL of linker solution and stirred overnight at room temperature. The resulting UiO-66 powder was collected by centrifugation and dried at 80 °C.

For the physical mixture, 1000 mg of P­(VDF-TrFE) was dissolved in 10 mL DMF in a 20 mL vial, followed by the addition of 97 mg UiO-66 (corresponding to 91 wt % P­(VDF-TrFE) and 9 wt % UiO-66). The mixture was sonicated for 4 h and then centrifuged at 10,000 rpm. The supernatant was discarded, and the obtained solution was then washed with DMF and methanol via centrifugation. Then, the solution was dried in the oven overnight, and the dried P­(VDF-TrFE)-UiO-66 physical mixture was collected.

#### In Situ Composites

For the in situ composites, 5 mL of node solution was combined with 5 mL of linker + polymer solution (polymer wt % of 56 and 91 wt %) and stirred overnight. The mixture was then centrifuged at 10,000 rpm. The supernatant was discarded, and the pellet was washed with DMF and methanol via centrifugation. The mixture was then dried in the oven overnight to obtain in situ P­(VDF-TrFE)-UiO-66 composite powder.

### Grazing Incidence X-ray Diffraction (GIXD)

GIXD was performed at beamline 11–3 of the Stanford Synchrotron Radiation Lightsource at the SLAC National Accelerator Laboratory using a fixed beam energy of 12.7 keV. A Rayonix MX225 CCD area detector was used to record the two-dimensional (2D) diffraction patterns with a sample-to-detector distance of 316 mm. MATLAB GUI was used to conduct fast azimuthal integration to extract 1D diffraction patterns from 2D GIXD images.

### Scanning Electron Microscope (SEM) and Energy Dispersive X-ray Spectroscopy (EDS)

SEM and EDS images were obtained using a FEI Quanta 650 scanning electron microscope. The electrons were accelerated at 5 kV. A spot size of 4 and a working distance of 10 mm were used to generate the images. Secondary electrons detected by the Everhart-Thornley detector (ETD) provided information on the surface topology, while the characteristic X-rays detected by the energy-dispersive X-ray spectroscopy detector provided elemental mapping of the samples. For EDS analysis, an accelerating voltage of 15 kV and a spot size of 5.5 were used to optimize the resolution of elemental mapping. Before SEM and EDS measurements, the thin film samples were sputter-coated with a gold/palladium layer using a Cressington sputter coater.

### Fourier-Transform Infrared Spectroscopy (FTIR)

FTIR spectra were obtained by using a PerkinElmer 400 FTIR spectrometer with an attenuated total reflectance (ATR) accessory, operating at a resolution of 1 cm^–1^ in the range of 4000 cm^–1^–700 cm^–1^. The thin film was placed inverted on top of the diamond, and pressure was applied using the lever arm of the FTIR to improve contact with the diamond.

### Differential Scanning Calorimetry (DSC)

DSC analysis of the polymer-MOF composites was performed using a TA Instruments DSC 2500. The composite films were first prepared via solution shearing and subsequently annealed at 130 °C for 2 h. After annealing, the films were removed from the substrate, and 5–10 mg of each sample was loaded into Tzero pans and sealed with Tzero hermetic lids. The samples were then heated and cooled between 50 and 150 °C at a rate of 10 °C/min.

### Brunauer–Emmett–Teller (BET) Analysis

The Micromeritics ASAP2020 Surface Area and Porosity Analyzer was used to measure the BET surface area. The samples were degassed at 80 °C for 12 h. Nitrogen adsorption isotherms were obtained at 77 K, and the BET theory was applied to the data between 0.01 and 0.05 relative pressure to obtain the BET surface area.

### Profilometry

Bruker DektakXT Stylus Profilometer was used to measure the thickness of the films. Before taking the measurements, a thin strip of material was cut from the center of the film using a razor blade. One mg stylus force, 10 μm/s scan speed, and 1000 μm of length (600 μm of film and 400 μm of bare substrate) were used for the measurement.

### Piezoelectricity Measurement

A customized setup was used to measure the piezoelectricity and apply identical forces to the deposited film. The experimental setup comprised a sample holder, pneumatic cylinders, electrically controlled solenoid valves (HB-2A0A-12), and electrical controllers. The solenoid valve was connected to electrical controllers to apply 12 V-based air gating, and an N_2_ source was used to apply identical and periodic force to the film. An oscilloscope (KEYSIGHT DSO-X 3024T) was used to measure microsecond pulse output and peak voltage. A SingleTact force sensor was used to measure the amplitude of the applied force. The contact area of the force applicator was approximately 0.5 cm × 0.5 cm, in the shape of a flat circular tip, enabling uniform and perpendicular stress distribution across the sample surface.

### Thermal Conductivity Measurement

Time-domain thermoreflectance (TDTR)^43–45^ was employed to measure the thermal properties of the polymer-MOF composites. TDTR is an optical, noncontact, laser-based, pump–probe measurement technique that measures the temporal decay of the pump-induced modulated temperature rise on the surface of a sample and relates this decay to the thermal conductivity of the material under the surface. In this method, the output of an 80 MHz, subpicosecond Ti: Sapphire laser is divided into separate pump and probe paths. The pump pulses are electro-optically modulated to a frequency of 8.4 MHz and then focused onto the sample. The probe pulses pass through a mechanical delay stage, which temporally delays the probe pulses relative to the pump pulses. The pump and probe beams are focused onto the sample using a 10× objective lens, resulting in focused 1/e^2^ pump and probe radii of ∼19 μm and ∼11 μm, respectively. The reflected probe beam is sent to a balanced photodetector, which measures the thermoreflectance of the film as a function of the pump–probe delay time. Fitting the measured thermal decay to an analytical solution to the cylindrical heat equation allows the determination of the thermal conductivity of the MOFs of interest.

As the surface of the polymer-MOF composites studied in this work was too rough to facilitate TDTR measurements, we employed a bidirectional technique^44,46–48^ where the MOF composites were deposited on glass slides coated with 80 nm of aluminum. TDTR measurements were then conducted through the glass substrate, and the Al surface in contact with the glass slide was used as the TDTR transducer. In our analysis of the TDTR data, we monitor the ratio of the in-phase to out-of-phase voltage of the lock-in amplifier and fit this data as a function of time from 300 ps to 5.5 ns to the solution of the aforementioned cylindrically symmetric heat equation. We assume literature values for the heat capacity of the Al transducer and glass substrate and determine the thermal conductivity of the Al transducer using the Wiedemann–Franz law applied to electrical resistivity measurements. We measure the thermal boundary conductance between the Al transducer and glass substrate, in addition to the thermal conductivity of the glass, using TDTR on a control sample. The volumetric heat capacities of the MOF composite were found from DSC and elsewhere.^49–51^ Neumann-Kopp rule was used to calculate the heat capacities of UiO-66 doped with varying amounts of P­(VDF-TrFE).

### Cyclic Voltammetry (CV) and Electrochemical Impedance Spectroscopy (EIS)

Films were fabricated on an Indium Tin Oxide (ITO)-coated glass slide. CV and EIS measurements of the modified layers were carried out using an electrochemical workstation (CH920C, CH Instruments, Austin, Texas) to gauge the electrochemical performance of the developed composite. All the experiments were carried out using a 2 cm^2^ ITO as the working electrode and an Ag/AgCl (3 M KCl) as the reference electrode. The counter electrode used was a Pt wire.

### Solid-State NMR Spectroscopy

^1^H and ^13^C solid-state NMR spectra were acquired using a Bruker 600 MHz Avance III spectrometer and a 4 mm magic angle spinning (MAS) HFX probe configured to ^1^H–^13^C or ^19^F–^13^C configurations at the National High Magnetic Field Laboratory (NHMFL). Samples were packed into Bruker 4 mm zirconia rotors with vespel caps and spun at 10 kHz MAS frequency. Radiofrequency (rf) pulses (π/2 and π) on ^1^H and ^13^C channels used 100 kHz and 68 kHz rf powers, respectively. Cross-polarization (CP) MAS experiments were performed with spinlock powers of 58 kHz and 68 kHz rf powers on ^1^H/^19^F and ^13^C channels, respectively, and spinlock pulse lengths of 1 ms were used. The ^1^H/^19^F spinlock pulse was ramped from 80 to 100% amplitude. SPINAL-64 heteronuclear decoupling was applied at 70 kHz rf. 1D ^1^H→^13^C CPMAS spectra were obtained using the total suppression of sidebands (TOSS) technique.^52^ ^1^H and ^13^C NMR spectra were referenced with respect to Adamantane (^1^H at 1.72 ppm and ^13^C at 37.777 ppm). 2D ^13^C­{^1^H} heteronuclear correlation spectra were performed with the eDUMBO homonuclear decoupling applied during the ^1^H *t*_1_-evolution period: 32 μs eDUMBO-1_22_ pulses^53^ at 100 kHz rf were used, and the F_1_ spectral width was scaled by a scaling factor of 1.64–1.70.

## Results and Discussion

Initially, polymer-MOF solutions with different concentrations (Table S1) of P­(VDF-TrFE) were synthesized by mixing the Zr-oxo node solution with the linker and polymer solution (Figure 1c). The solutions were then fabricated into thin films via solution shearing at 90 °C with a blade speed of 0.05 mm/s. Following deposition, the films were annealed at 130 °C for 2 h.

The diffraction patterns of the polymer-MOF composite thin films were compared with the diffraction pattern of the P­(VDF-TrFE) control and the simulated pattern of UiO-66.^8^ The GIXD data (Figure 2a) confirms the synthesis of UiO-66 MOF crystals along with the presence of the β-phase crystalline domains of the P­(VDF-TrFE) polymer. Diffraction peaks at q values of 0.52 and 0.60 Å^–1^ correspond to the scattering from the (111) and (002) crystal planes of UiO-66, respectively.^54^ Similarly, the diffraction peak at the q value of 1.40 Å^–1^ represents diffraction from the (110/200) crystal plane of the piezoelectric β-phase of P­(VDF-TrFE).^55^ As seen in Figure 2a, no β-phase crystalline peak is seen for composites made using a lower polymer concentration (56 wt %). Previous studies have shown that the crystallization of P­(VDF-TrFE) in the composite can be hindered by increasing the concentration of nanoparticles such as MOFs, as the high concentration of MOF particles interrupts the growth of polymer crystals.^56,57^ As seen from two-dimensional GIXD patterns (Figure S1), solution shearing did not yield oriented crystals of UiO-66 and P­(VDF-TrFE).

**Figure 2:**
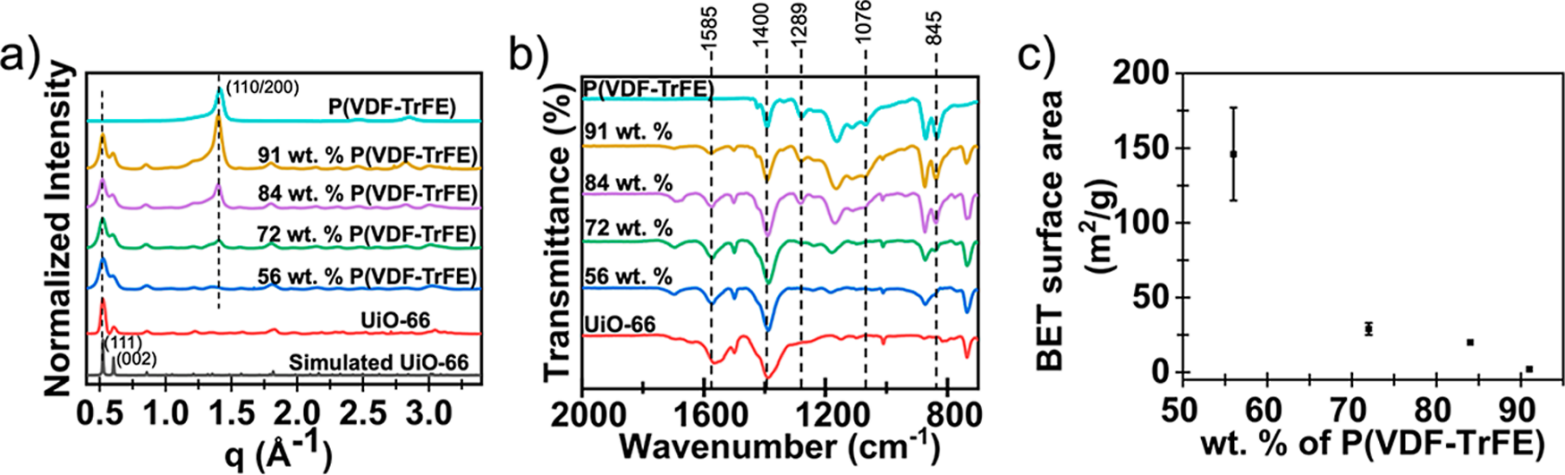
(a) GIXD patterns, (b) FTIR analysis, and (c) BET surface area for composite thin films with different relative wt % of P­(VDF-TrFE) in the P­(VDF-TrFE)-UiO-66 composite films.

To confirm the presence of characteristic functional groups from both the MOF and polymer components, FTIR analysis was performed. As shown in Figure 2b, the absorption peaks of the polymer-MOF composites match those of UiO-66 and P­(VDF-TrFE), respectively. The absorption bands at 845 cm^–1^, 1076 cm^–1,^ and 1289 cm^–1^ represent the β-phase of P­(VDF-TrFE). The peaks at 845 cm^–1^ represent CF_2_ stretching, and 1076 cm^–1^ represents CH_2_ wagging and C–C stretching of the polymer chain.^58^ Similarly, the peaks at 1585 cm^–1^ and 1400 cm^–1^ are due to the in- and out-of-phase stretching of the carboxylate groups in UiO-66.^59^ Consistent with the GIXD results, no absorption band for the β-phase (845 cm^–1^) is seen for the composite created with 56 wt % P­(VDF-TrFE).

We performed DSC analysis to validate the findings from GIXD and FTIR. As shown in Figure S2, the heating curves for P­(VDF-TrFE) and composites display two characteristic thermal events. The first endothermic peak (at 122 °C for P­(VDF-TrFE)) corresponds to the ferroelectric-to-paraelectric phase transition (Curie temperature), while the second peak (at 145 °C for P­(VDF-TrFE)) represents the melting of the crystalline phase.^60^ Both peak intensities decrease as the polymer concentration in the composite decreases. Additionally, increasing the MOF content leads to a reduction in both Curie and melting temperatures. Notably, while no crystalline β-phase signal was seen in either GIXD or FTIR results for the 56 wt % composite, the presence of the melting peak at 140 °C (Figure S2) indicates the presence of crystalline polymer in the composite. However, the significantly lower peak intensity for the 56 wt % composite, compared to the P­(VDF-TrFE) control and the 91 wt % composite, suggests a substantially reduced crystalline fraction at this composition.

To gauge the porosity of the composite films, Brunauer–Emmett–Teller (BET) analysis was done using the N_2_ adsorption isotherm to calculate the specific surface area of the composites (Figure 2c). The BET surface area of the pristine UiO-66 is ∼1200 m^2^/g.^61^ As shown in Figure 2c, the specific surface area of the MOF decreases with the incorporation of P­(VDF-TrFE). The composite with 56 wt % polymer showed a surface area of 146 m^2^/g, while the surface area of the composite with 91 wt % polymer was 2 m^2^/g, suggesting that at high polymer concentrations, the pores of MOF are completely blocked by the excess polymer.

The surface morphology of the thin films was characterized using SEM (Figure 3 and Supporting Information Figures S3–S7). The pure UiO-66 film displays cracks (Figure S3), indicating incomplete substrate coverage by the UiO-66 crystals. This result aligns with Jung et al.’s findings, which showed that multiple passes of solution shearing are needed to achieve a fully covered UiO-66 film.^30^ The addition of P­(VDF-TrFE) eliminates these cracks (Figures S4–S7), resulting in better film coverage. The surface coverage analysis (Figures S8–S12) shows that coverage increased from 70% for the UiO-66 film to 100% for the 84 and 91 wt % P­(VDF-TrFE)-UiO-66 composite films, indicating that polymer incorporation significantly enhances film coverage. Furthermore, as the concentration of P­(VDF-TrFE) is increased, spherical crystals of UiO-66 covered and interconnected by polymer strands are formed (Figures 3a,b, S4 and S5). With a further increase in polymer concentration (84 and 91 wt %), we observe small patches of polymer-dominated regions where crystals of UiO-66 are present sparingly (Figures 3c,d, S6 and S7). Additionally, the presence of P­(VDF-TrFE) and UiO-66 throughout the films was confirmed by the presence of fluorine (F) and zirconium (Zr) elemental signatures, respectively (Figure 3), which were obtained by using EDS. In addition to the SEM and EDS analysis, the thickness of the composite films was measured using a profilometer, and the results are summarized in Table S2. The composite containing 91 wt % P­(VDF-TrFE) had the highest film thickness of 5.7 ± 1.3 μm, whereas the P­(VDF-TrFE) control film measured 2.1 ± 0.6 μm in thickness.

**Figure 3:**
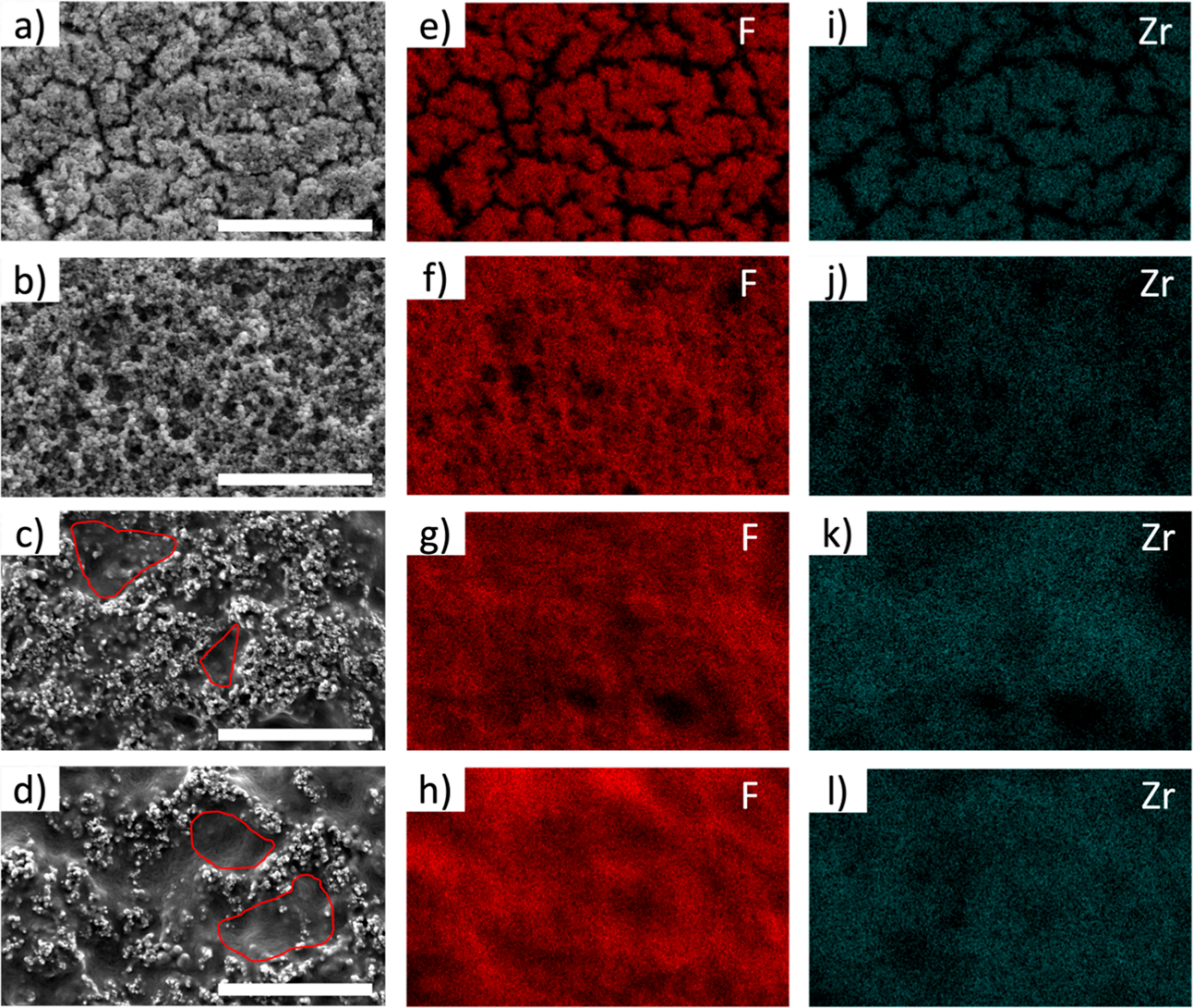
(a–d) SEM images, (e–h) fluorine elemental mapping (i–l) zirconium elemental mapping of solution sheared P­(VDF-TrFE)-UiO-66 films with 56, 72, 84, and 91 wt % P­(VDF-TrFE), respectively (scale = 10 μm).

To probe the penetration of P­(VDF-TrFE) into the pores of UiO-66, we applied solid-state NMR to neat UiO-66, neat P­(VDF-TrFE), a physical mixture of UiO-66 and P­(VDF-TrFE), 56 wt %, and 91 wt % P­(VDF-TrFE)-UiO-66 composites. Figure S13 shows ^1^H MAS NMR spectra of these samples: signals corresponding to P­(VDF-TrFE) appear near 2–5 ppm, UiO-66 shows characteristic aromatic signals near 6.5–7.5 ppm, and signals corresponding to DMF and acetate near 1–2 ppm.^62^ Comparison of ^1^H NMR spectra of 91 wt % P­(VDF-TrFE) with the neat UiO-66 shows a clear increase in line widths of the aromatic signals, suggesting interaction of the polymer with the MOF linkers, similar to previous observations.^20^ Furthermore, the ^1^H longitudinal relaxation times (*T*_1_) of the MOF and polymer signals in the composite were similar (*T*_1_ ∼ 2.4 s), likely due to significant ^1^H–^1^H spin diffusion between the protons on UiO-66 and P­(VDF-TrFE). As a control, we measured the ^1^H *T*_1_ values of a physical mixture of UiO-66 and P­(VDF-TrFE), which were significantly different (Figure S13).

Figure 4 shows ^1^H→^13^C cross-polarization (CP) magic angle spinning (MAS) spectra of the five samples studied. With the 56 wt % P­(VDF-TrFE) composite film we observe very low signal intensities corresponding to P­(VDF-TrFE) in both ^1^H and ^13^C NMR spectra (Figures 4 and S13). Taken together with the IR, DSC, and GIXD results above, these observations suggest minimal inclusion of P­(VDF-TrFE) in the composite with a 56 wt % initial fraction of P­(VDF-TrFE) during synthesis. With the 91 wt % P­(VDF-TrFE) composite, ^13^C signals from P­(VDF-TrFE) can be clearly distinguished from UiO-66, but these signals do not dominate the spectrum. Cumulatively, these observations suggest that the actual incorporation of polymer in the MOF could be lower than expected using the initial solution stoichiometry. Comparison of the ^13^C line widths in the CP spectra shows a slight increase in line width for the aromatic signal at ∼129 ppm and the carboxylate signal at ∼170 ppm, along with shifts in these peak positions by +0.4 ppm in the 91 wt % P­(VDF-TrFE) composite, in comparison to the neat UiO-66 and physical mixture samples (Figure S14). The observations made from ^1^H and ^13^C NMR spectra suggest that the protons of UiO-66 and P­(VDF-TrFE) are in close spatial proximity. However, further analysis is necessary to establish the extent and distances of these contacts.

**Figure 4:**
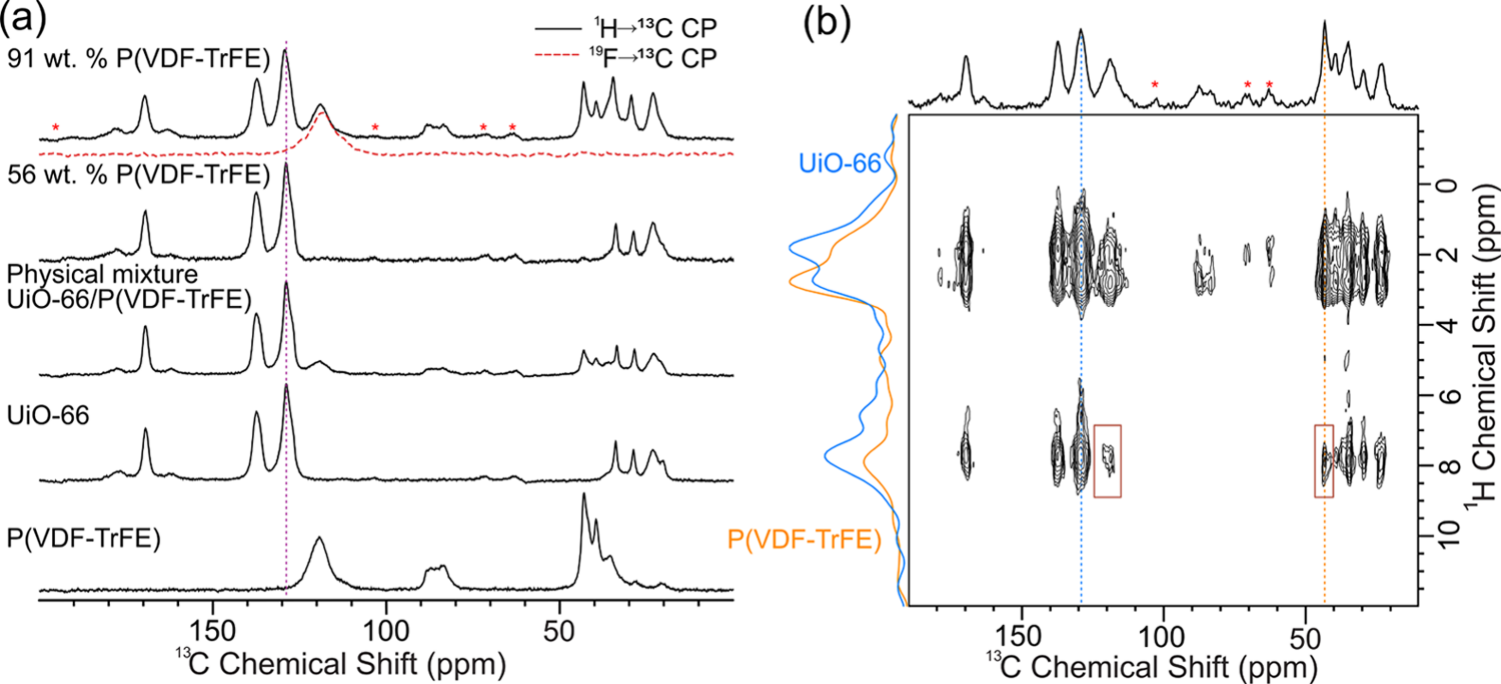
(a) ^1^H→^13^C CP MAS solid-state NMR spectra of (top to bottom) 91 and 56 wt % P­(VDF-TrFE) composite films, physical mixture of UiO-66 and P­(VDF)-TrFE, and neat UiO-66 and P­(VDF-TrFE). ^19^F→^13^C CP MAS spectrum of 91 wt % P­(VDF-TrFE) is also shown (red). (b) 2D ^13^C­{^1^H} heteronuclear correlation solid-state NMR spectrum of 91 wt % P­(VDF-TrFE) with 25 ms ^1^H–^1^H spin diffusion. Cross peaks showing the close proximity of UiO-66 and P­(VDF-TrFE) are highlighted in brown. ^1^H slices extracted at the indicated ^13^C chemical shifts of 43 ppm (yellow, P­(VDF-TrFE)) and 129 ppm (blue, UiO-66) are shown. Spinning sidebands are indicated with red asterisks (*).

To evaluate the extent of mixing, we acquired 2D ^13^C­{^1^H} heteronuclear correlation solid-state NMR spectra with a ^1^H–^1^H spin diffusion period prior to the ^1^H→^13^C CP transfer. Figure 4b shows a 2D ^13^C­{^1^H} heteronuclear correlation (HETCOR) solid-state NMR spectrum of the 91 wt % P­(VDF-TrFE) composite film obtained with a 25 ms ^1^H–^1^H spin diffusion period. Clearly, ^1^H–^1^H spin diffusion is present between the UiO-66 and P­(VDF-TrFE) signals, resulting in the highlighted cross peaks. These cross peaks are absent without any spin diffusion period and are also absent in the physical mixture sample (Figure S15). These observations support the conclusion that P­(VDF-TrFE) is incorporated inside the pores of UiO-66 in the 91 wt % P­(VDF-TrFE) composite.

However, the observed cross peaks are only moderately intense, and ^1^H traces extracted from the 2D spectrum at shifts corresponding to UiO-66 (129 ppm) and P­(VDF-TrFE) (43 ppm) show different magnetization profiles. The absence of identical magnetization profiles indicates partial mixing of the polymer and the MOF.^20^ We obtained a series of 2D ^13^C­{^1^H} HETCOR spectra at a series of mixing times, which showed a maximum intensity of the cross peaks at spin diffusion times greater than 25 ms (Figure S16). In analogous studies, Schmidt-Rohr and co-workers observed homogeneous mixing at 2 ms mixing times with UiO-66/PEO mixed matrix membranes. In contrast, they observed a much slower spin diffusion (weak cross peak at 50 ms mixing) with UiO-66/PVDF, which was proposed to have a surface coating model.^20^ Here, we observe a moderately intense cross peak (∼21% of the signal intensity of the P­(VDF-TrFE) signal before spin diffusion) at a 25 ms mixing time, suggesting estimated spatial proximities that are likely intermediate between the previously proposed 1–2 nm pore infiltration model and the surface coating model.^20^ Based on all these observations, we propose that there is a partial penetration of P­(VDF-TrFE) inside UiO-66, with the possibility of a significant fraction of the polymer strands present near but outside the pores of the MOF.

The piezoelectric response of the polymer-MOF film was measured using a customized force applier to apply a force of 35 N to the composite film. When a mechanical force is applied to a P­(VDF-TrFE) film, its β-phase crystalline domains undergo strain, causing a dynamic change in net polarization. This time-dependent polarization change leads to charge displacement and generation of an electric potential across the film. The magnitude of the voltage response is directly influenced by the degree of crystallinity and orientation of the β-phase domains within the composite. Therefore, the periodic spike in voltage was studied in comparison to the wt % of P­(VDF-TrFE) in the polymer-MOF composite films.

The voltage output of the films was measured across different proportions of P­(VDF-TrFE) (Figure 5a) to showcase the piezoelectric performance of the films. A statistical analysis of the piezoelectric performance of the composite films, deposited via solution shearing and drop casting, was conducted. The mean output voltage was found to be directly proportional to the weight percentage of P­(VDF-TrFE) in the composite (Figure 5b), reaching a maximum of 9.1 V at 91 wt % P­(VDF-TrFE). The enhancement in the piezoelectric voltage output is primarily due to the increased formation of the crystalline β-phase of P­(VDF-TrFE) as confirmed by GIXD, FTIR, and DSC analysis. The standard deviations were relatively low, indicating a general uniformity in the piezoelectric performance across most compositions. The uniformity in the piezoelectric performance suggests effective dispersion and integration of the UiO-66 and P­(VDF-TrFE) in composite thin film facilitated by the solution shearing technique. Conversely, when fabricated using the drop-cast method, the same composites showed lower average values and higher standard deviation in all tested cases (Figure 5b). The composite films also demonstrated notable sensitivity, with the 91 wt % P­(VDF-TrFE) film showing a sensitivity of 0.26 V/N (Table S3). These values are comparable to those reported in the literature for P­(VDF-TrFE) films cast using techniques such as electrospinning (Table S4).^24,39,63,64^ The appreciable piezoelectric performance and low variability in output voltage indicate that the solution shearing method is a viable technique for fabricating uniform, high-performance piezoelectric polymer-MOF composite thin films.

**Figure 5:**
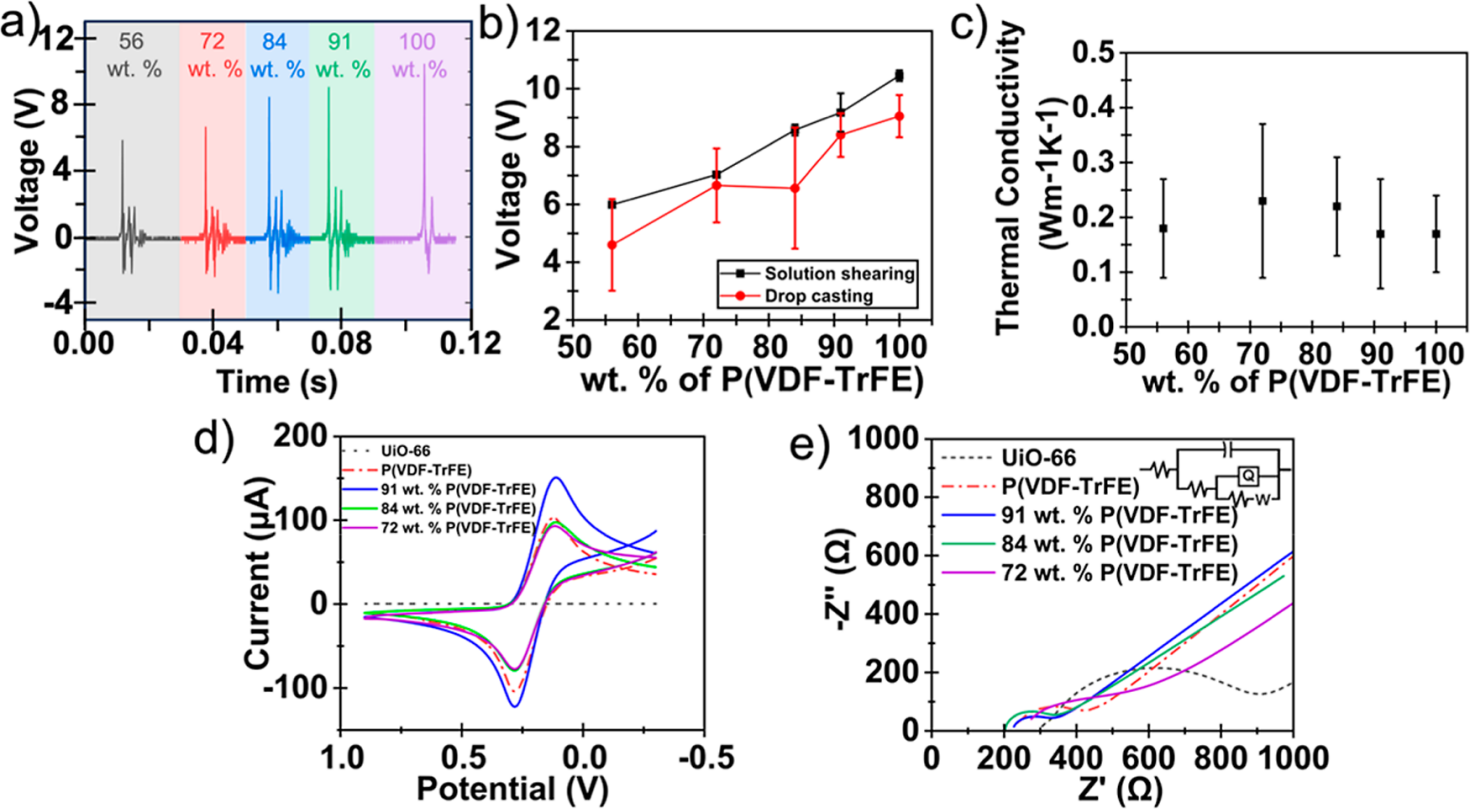
(a) Pulse response for an applied force with different P­(VDF-TrFE) amounts in P­(VDF-TrFE)-UiO-66 films fabricated by the solution shearing method (insets show the wt % of P­(VDF-TrFE) in the composite films). (b) Average piezoelectric voltage output in P­(VDF-TrFE)-UiO-66 composite films fabricated by solution shearing and drop casting method. The plot includes the experimental measured result of mean voltages and standard deviations across different compositions of polymer and MOF in the composite films. (c) Average thermal conductivity of the composite films with varying wt % of P­(VDF-TrFE). (d) The cyclic voltammograms (scan rate of 0.1 V/s) and (e) corresponding electrochemical impedance spectra (EIS) in 5 mM Ke_3_Fe­(CN)_6_ in 0.1 M KCl. The inset shows the equivalent circuit that was used to fit the EIS spectra of composite films.

Understanding the thermal behavior of composite films is crucial for evaluating their suitability in sensors and electronic devices, where thermal conductivity significantly impacts performance and reliability. Since the infiltration of MOF pores with guest molecules has been shown to significantly impact the thermal transport properties of composites, we examined how the incorporation of P­(VDF-TrFE) with UiO-66 affects the thermal conductivity of our composite films.^48^ The thermal conductivities of the P­(VDF-TrFE)-UiO-66 thin film series were measured using time-domain thermoreflectance (TDTR). As shown in Figure 5c, the thermal conductivities of the polymer-MOF thin films are similar within error, with values between 0.23 ± 0.14 W m^–1^ K^–1^ and 0.17 ± 0.10 W m^–1^ K^–1^. For pure P­(VDF-TrFE) film, a thermal conductivity of 0.17 ± 0.07 W m^–1^ K^–1^ was measured, which is in agreement with values reported in the literature.^51^ The observed trend suggests that increasing the P­(VDF-TrFE) content in the composite does not result in a significant change in thermal conductivity, as both P­(VDF-TrFE) and UiO-66 exhibit similar thermal conductivities. The low thermal conductivities of the films can be attributed to high vibrational scattering in the amorphous regions of the material, a common phenomenon found in semicrystalline materials.^65–67^ In MOF complexes, the vibrational scattering is partially due to the large difference in mass between the metallic nodes and organic linkers, as well as due to the increase of scattering in the pores.^47,48,68,69^

Furthermore, electrochemical impedance spectroscopy (EIS) and cyclic voltammetry (CV) were used to determine the resistance (for conductivity calculation), electrochemical activity, and redox behavior of the film (Figure 5d,e). It can be seen from Figure 5d that the UiO-66 film did not show any response to CV in 5 mM Ke_3_Fe­(CN)_6_ due to the high resistance of ∼600 Ω that was shown in the EIS spectra (Figure 5e). The insulating nature of UiO-66 supports this observation. The P­(VDF-TrFE) layer showed a higher current response (anodic current *I*_a_ of 102.80 μA; Table S5) and lower resistance (200 Ω). The Δ*E* value of this modification (159.2 mV) was also low and could be due to the lower potential required for the redox of the ferricyanide anion. Among the different polymer wt %, the composite containing 91 wt % showed the lowest electron transfer resistance (100 Ω). This composite showed an anodic current *I*_a_ of 111.80 μA and a Δ*E* value of 150.9 mV. This slight decrease in resistance with the addition of UiO-66 could be attributed to the ability of open zirconium metal sites in UiO-66 to conduct anions, leading to improved transfer of anions (ferricyanide and ferrocyanide) across the films.^70^ However, when the amount of P­(VDF-TrFE) was reduced below 91 wt %, the electron transfer resistance of the composite was found to be higher due to the insulating nature of the UiO-66, which dominated the composite behavior at a lower wt % of P­(VDF-TrFE).

## Conclusion

In this study, we employed a meniscus-guided coating technique, ″solution shearing,″ to fabricate films of P­(VDF-TrFE)-UiO-66 polymer-MOF composites. Thin films with varying concentrations of polymer and MOF were fabricated to investigate the effects of polymer concentration on the crystallinity, surface morphology, piezoelectricity, and conductivity of the composite thin films. Using solution shearing, we were able to produce large area (∼1 in.^2^) thin films of P­(VDF-TrFE)-UiO-66 within minutes. We found that the addition of P­(VDF-TrFE) did not impact the crystallization of UiO-66 and enhanced the surface coverage of the film. Additionally, solid-state NMR spectroscopy provided further insight into the interaction between P­(VDF-TrFE) and UiO-66, revealing differences in polymer-MOF interactions between the composites formed via in situ MOF growth and those prepared as physical mixtures. The in situ composites exhibited stronger polymer-MOF interactions, with evidence of polymer chains infiltrating the pores of UiO-66. Furthermore, the addition of P­(VDF-TrFE) introduced piezoelectric properties into the composite. Among the composites, the composite with 91 wt % P­(VDF-TrFE) showed the highest piezoelectric performance with the maximum average output voltage of 9.1 V and sensitivity of 0.26 V/N. These values are comparable to those of other P­(VDF-TrFE) films fabricated using electrospinning techniques. The thermal conductivity of the composites was similar to both P­(VDF-TrFE) and UiO-66, while the electrical resistance of the films decreased with the addition of P­(VDF-TrFE). These results show that solution shearing is an effective technique for synthesizing polymer-MOF composite thin films, thus broadening the potential applications of polymer-MOF composites.

## Supplementary Material





## Notes

Calculation of ideal wt % of UiO-66 and P­(VDF-TrFE), along with the table showing the wt % of UiO-66 and P­(VDF-TrFE) in the composite films, two-dimensional GIXD patterns, DSC, SEM images of the composite films, tables showing the thickness of the solution sheared films, sensitivity (V/N), and comparison between piezoelectric performance reported in this study with values reported in literature, sensitivity plot for thermal conductivity analysis along with the ratio of in-phase and out-of-phase TDTR data vs time delay plot, table with anodic and cathodic currents and potentials derived from CV (PDF)
Raw solid-state NMR data is made available as a separate zip file (ZIP)

## ○

Department of Chemistry, University of Virginia, Charlottesville, Virginia 22904, United States
