# Supplementary material for: Fabrication of Piezoelectric Polymer and Metal–Organic Framework Composite Thin Films Using Solution Shearing
Source: ACS Appl Mater Interfaces. 2025 Jul 23;17(31):44899–909. doi: 10.1021/acsami.5c07907 (PMC12332846; doi:10.1021/acsami.5c07907)
Supplement: Supplementary file 1 [file am5c07907_si_001.pdf]

Supplemental Information

Fabrication of Piezoelectric Polymer and Metal-Organic Framework (MOF) Composite Thin Films using Solution Shearing

*Ankit Dhakal[1], Sangeun Jung[1], Byungjoon Bae[2], Ajith Mohan Arjun[2], Sean Robinson[1], Yongmin Baek[2], William T. Riffe[3], Emma M. Tiernan[4], Shubha Gunaga[8], Prince Verma[1], Meagan R. Phister[1], Madison Stone[1], Kevin H. Stone[6], Amanda Morris[7], Nathan S. Swami[2], Patrick E. Hopkins[3-5], Amrit Venkatesh[8†], Kyusang Lee[2] and Gaurav Giri[1]**

[1] Department of Chemical Engineering, University of Virginia, Charlottesville, VA, 22904-4746, USA

[2] Department of Electrical and Computer Engineering, University of Virginia, Charlottesville, VA, 22904-4746, USA

[3] Department of Materials Science and Engineering, University of Virginia, Charlottesville, VA 22904-4746, USA

[4] Department of Mechanical and Aerospace Engineering, University of Virginia, Charlottesville, VA 22904-4746, USA

[5] Department of Physics, University of Virginia, Charlottesville, VA 22904-4746, USA

[6] Stanford Synchrotron Radiation Lightsource, SLAC National Accelerator Laboratory, Menlo Park, CA 94025, USA

[7] Department of Chemistry, Virginia Tech, Blacksburg, VA 24061, USA

[8] National High Magnetic Field Laboratory, Florida State University, Tallahassee, FL 32310, USA

† Current address: Department of Chemistry, University of Virginia, Charlottesville, VA 22904, USA.

* Corresponding Author: gg3qd@virginia.edu

Calculation of ideal weight % for UiO-66 and P(VDF-TrFE) in composites:

Since, 355 μL of 70 % zirconium propoxide in 1-propanol was used to synthesize 11.355 ml of total node solution.

$355\ \mu L\ \times 0.7 \times \frac{1.044\ g}{ml} \times \frac{1\ ml}{1000\ \mu L} \times \frac{1\ mol}{327.5\ g}$ = 0.792 mmol of Zirconium propoxide

Since, 5 ml out of 11.355 ml node solution was used for the synthesis,

$0.792\ mmol \times \frac{5}{11.355} \times \frac{1\ mol\ UiO-66}{6\ mol\ Zirconium\ propoxide}$ = 0.058 mmol UiO-66

0.058 mmol UiO-66 $\times \frac{1\ mol}{1000\ mmol} \times \frac{1664.06\ g}{mol}$ = 0.097 g = 97 mg UiO-66

Table S1: Weight % of UiO-66 and P(VDF-TrFE) in solution used to create composites

Mass of P(VDF-TrFE)	Mass of UiO-66	Wt. % of P(VDF-TrFE)	Wt. % of UiO-66
1000 mg	97 mg	91	9
500 mg	97 mg	84	16
250 mg	97 mg	72	28
125 mg	97 mg	56	44

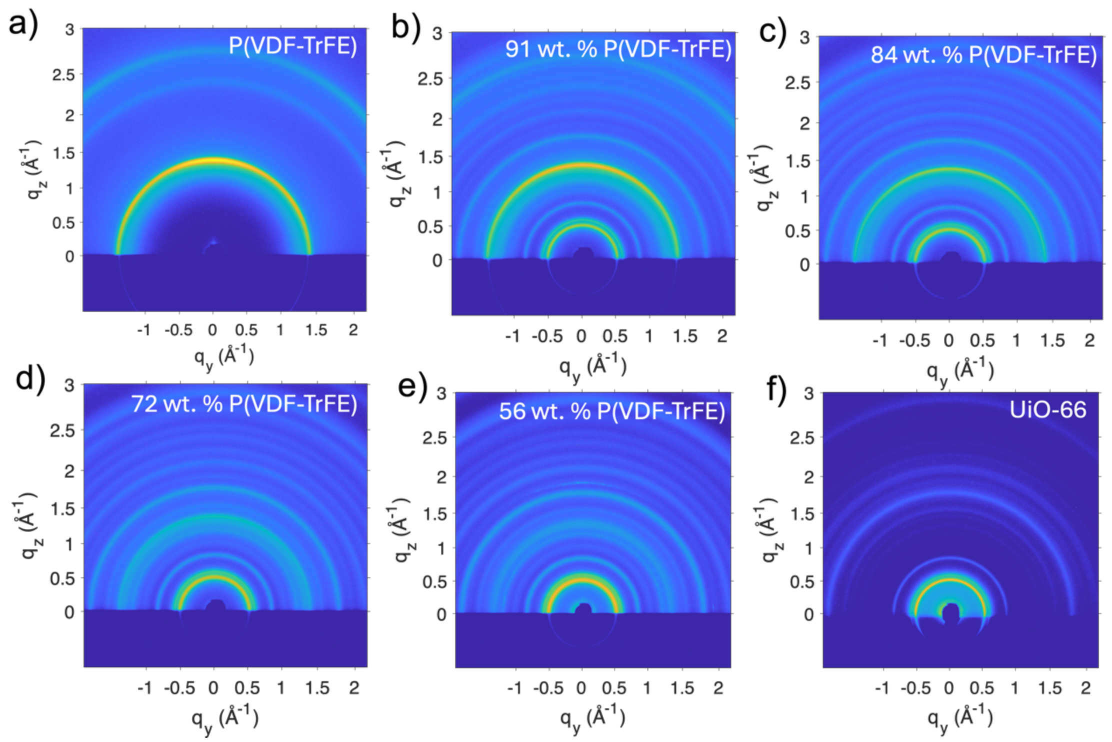

Figure S1: Two-dimensional grazing incidence X-ray diffraction (GIXD) patterns of solution sheared a) P(VDF-TrFE), b) 91 wt. % P(VDF-TrFE), c) 84 wt. % P(VDF-TrFE), d) 72 wt. % P(VDF-TrFE), e) 56 wt. % P(VDF-TrFE) and f) UiO-66 films.

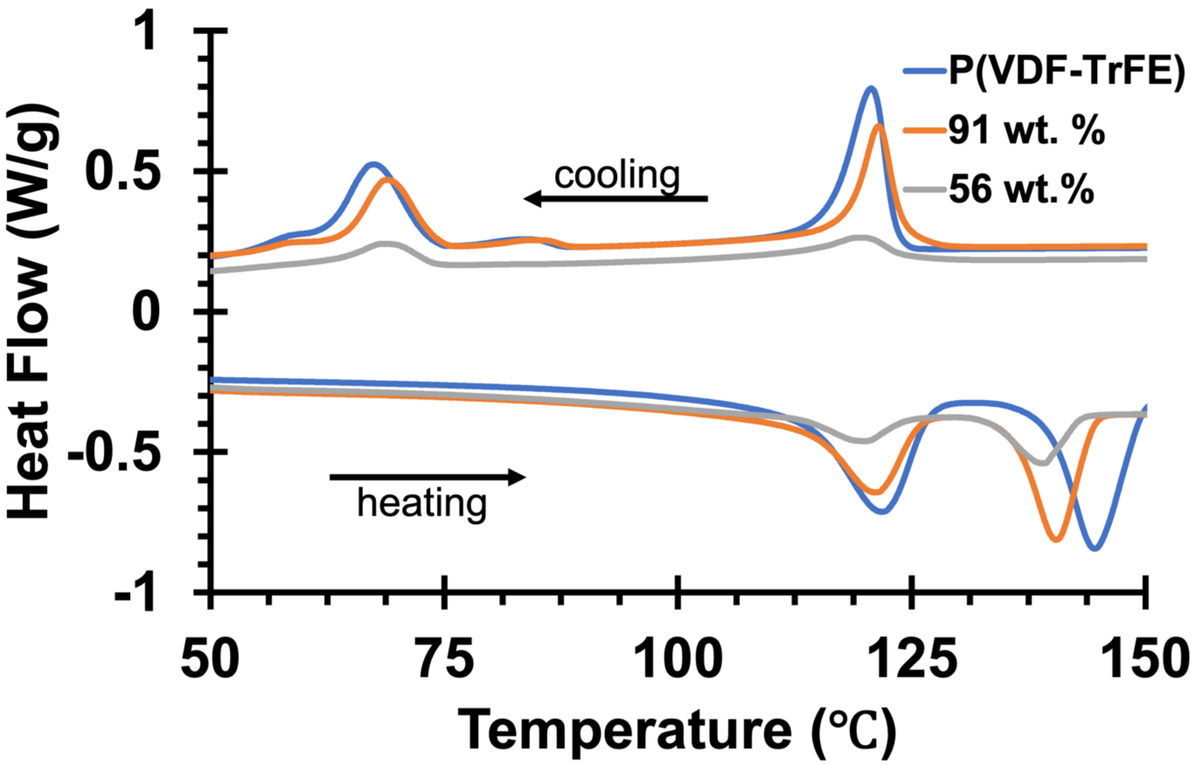

Figure S2: DSC analysis of P(VDF-TrFE)-UiO-66 composites

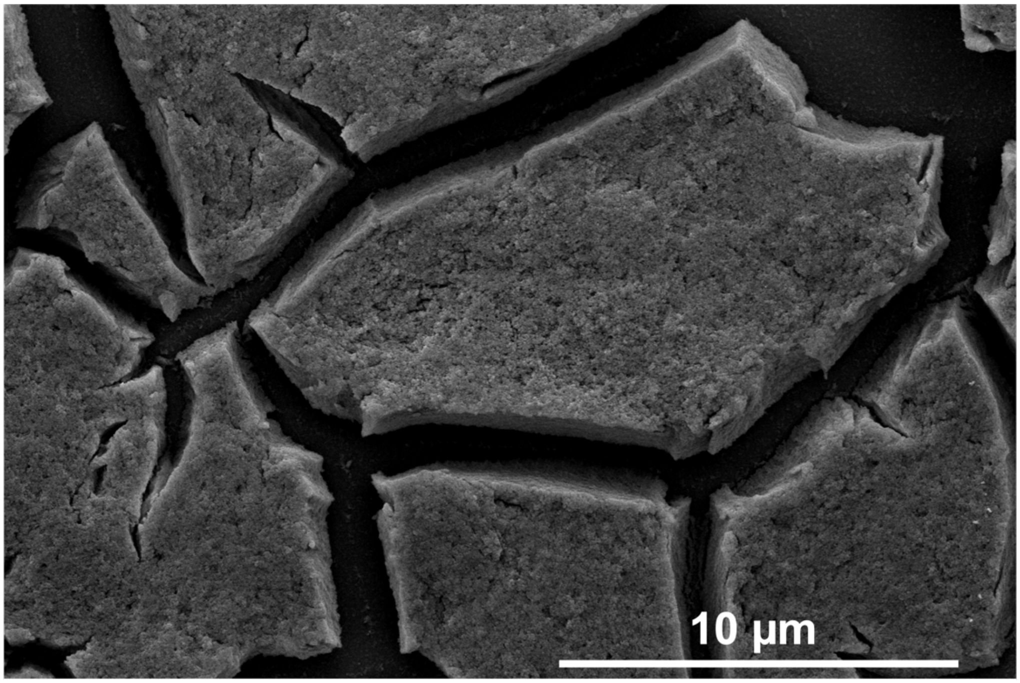

Figure S3: SEM image of UiO-66

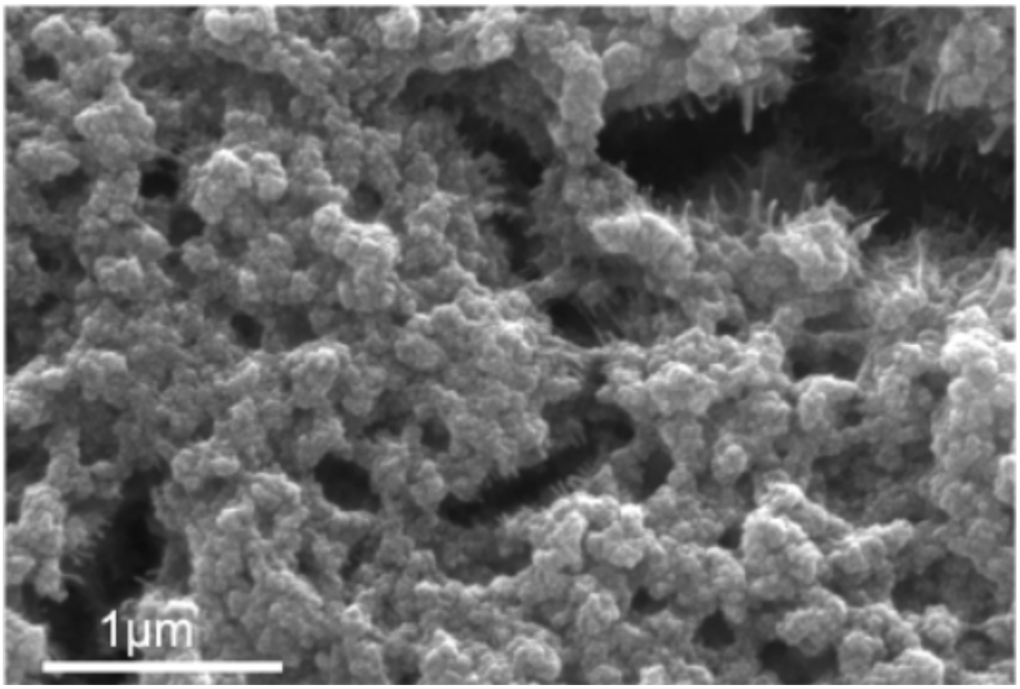

Figure S4: SEM image of 56 wt. % P(VDF-TrFE)-UiO-66

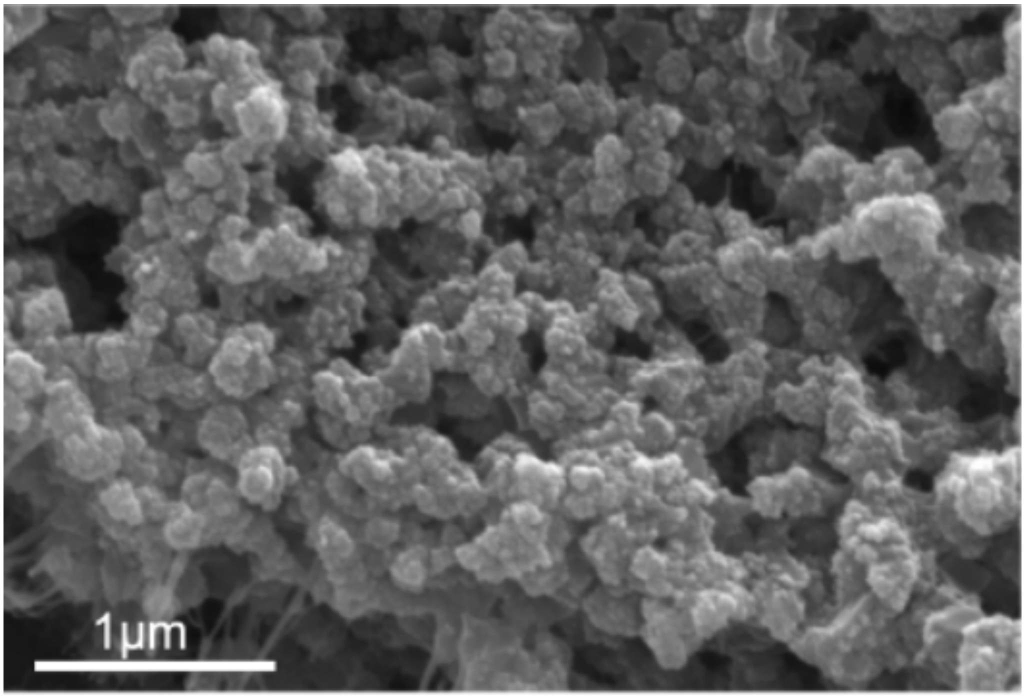

Figure S5: SEM image of 72 wt. % P(VDF-TrFE)-UiO-66

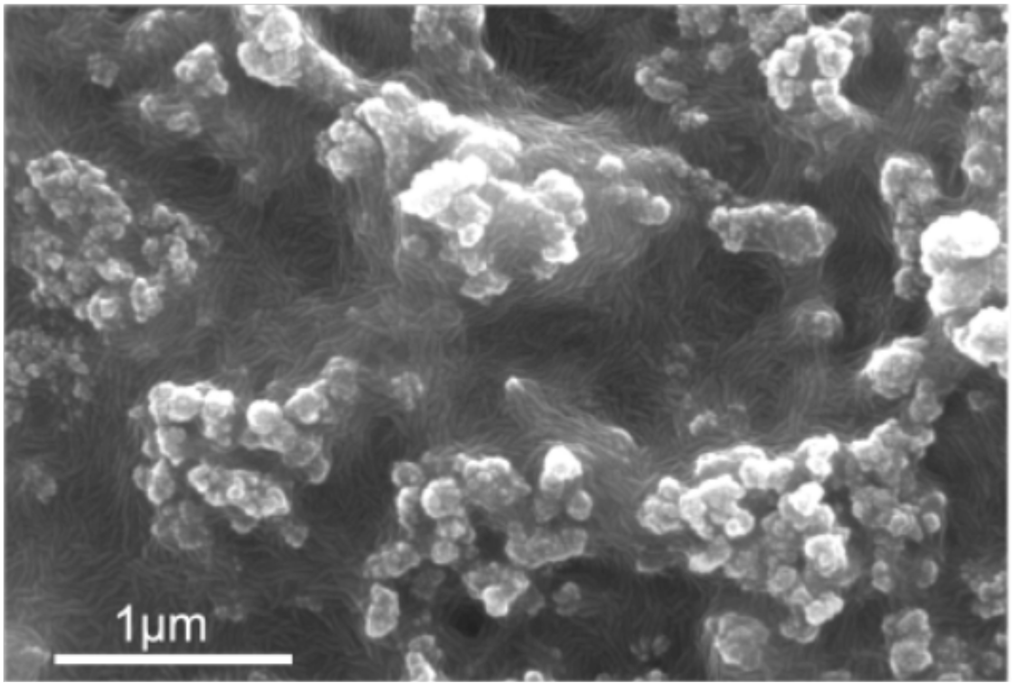

Figure S6: SEM image of 84 wt. % P(VDF-TrFE)-UiO-66

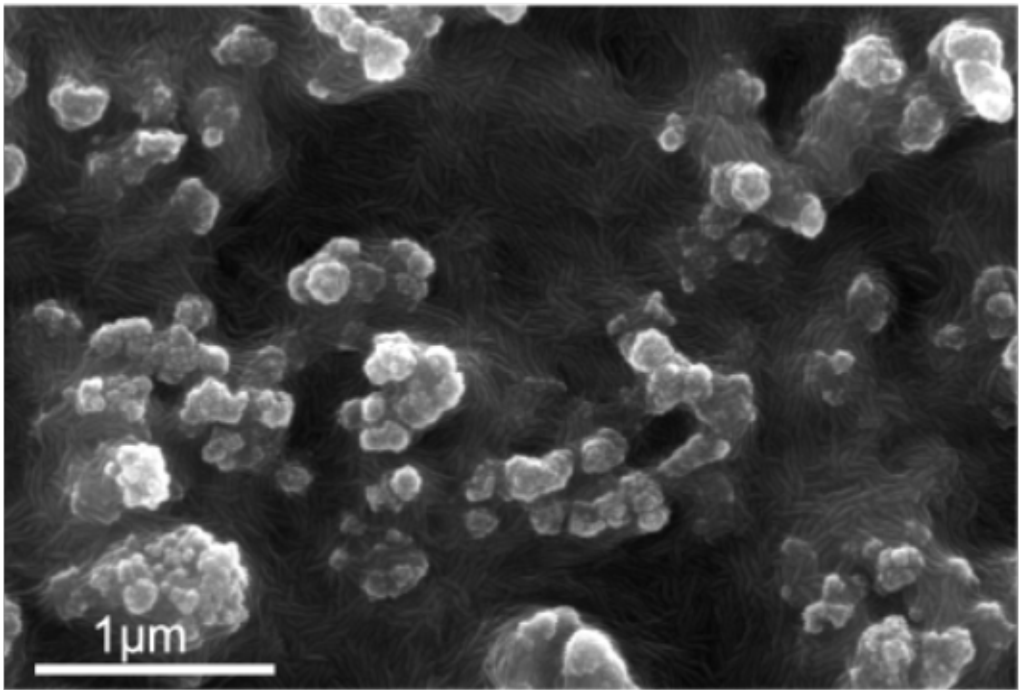

Figure S7: SEM image of 91 wt. % P(VDF-TrFE)-UiO-66

Surface Coverage Analysis:

The surface coverage of the films was analyzed using Fiji software. The images were converted into red and black images using the Image threshold function in the software, where the red area represents the film whereas the black area represents the substrate. The film coverage was then calculated using:

$$Film\ Coverage = \frac{Area\ covered\ by\ red\ color}{Total\ area}$$

Coverage = 0.70

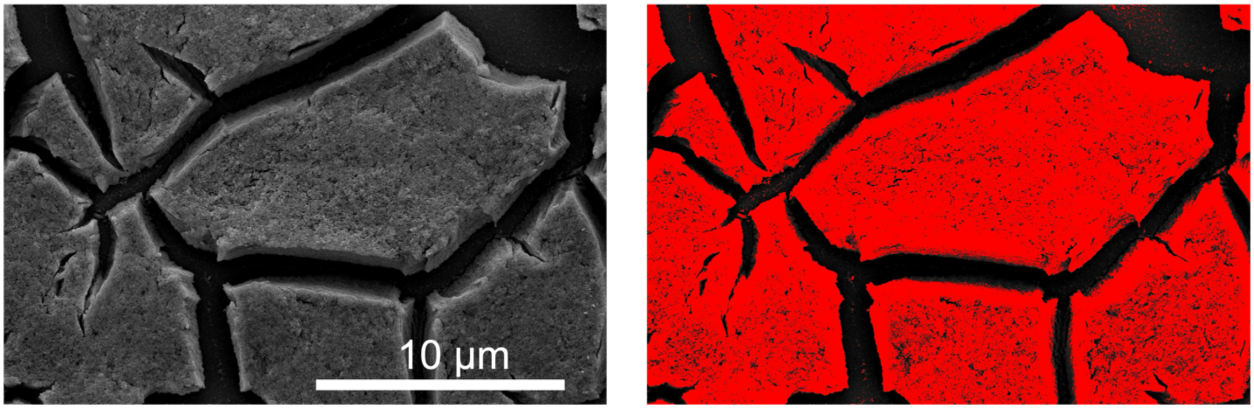

Figure S8: SEM image and its red-black threshold image of UiO-66 film

Coverage = 0.83

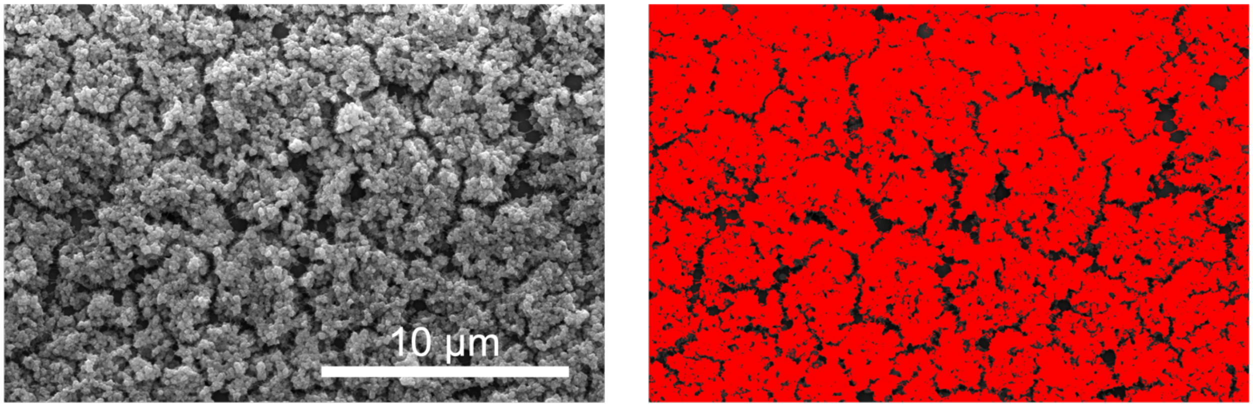

Figure S9: SEM image and its red-black threshold image of 56 wt. % P(VDF-TrFE)-UiO-66 composite film

Coverage = 0.90

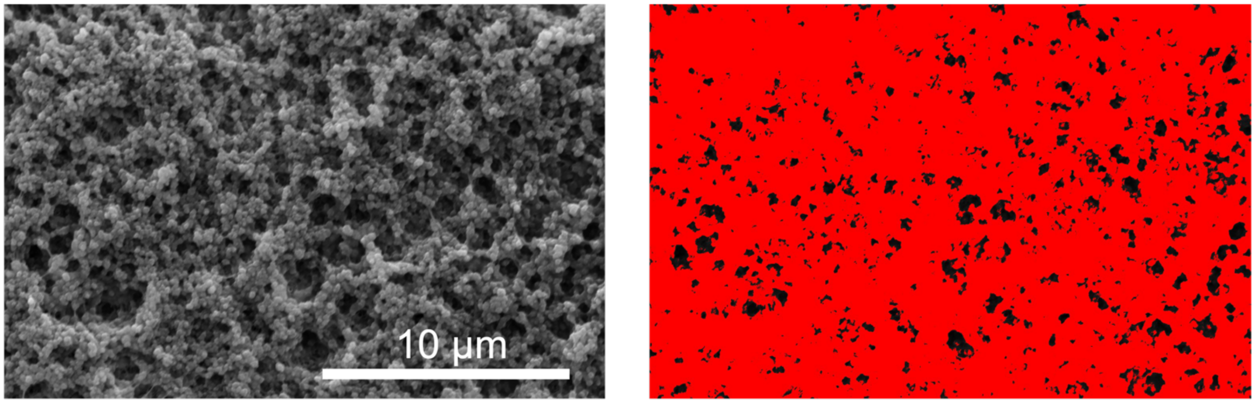

Figure S10: SEM image and its red-black threshold image of 72 wt. % P(VDF-TrFE)-UiO-66 composite film

Coverage = 1.00

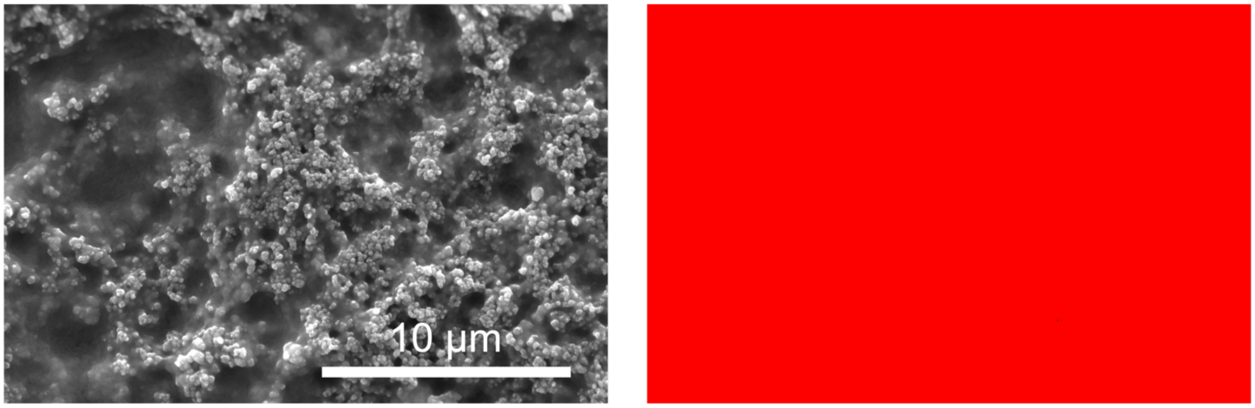

Figure S11: SEM image and its red-black threshold image of 84 wt. % P(VDF-TrFE)-UiO-66 composite film

Coverage = 1.00

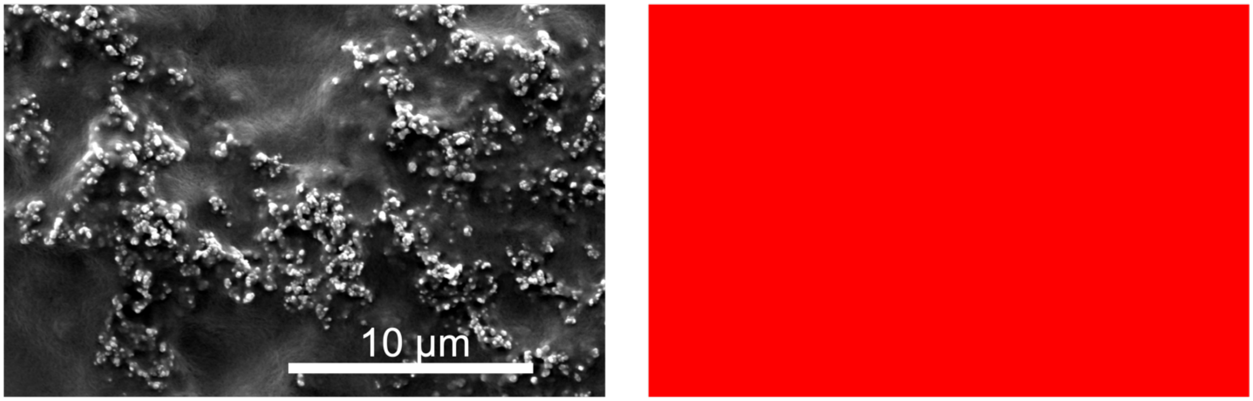

Figure S12: SEM image and its red-black threshold image of 91 wt. % P(VDF-TrFE)-UiO-66 composite film

Table S2: Thickness of the composite thin films measured using profilometer

Sample	Average thickness (μm)
P(VDF-TrFE)	2.1 ± 0.6
91 wt. % P(VDF-TrFE)	5.7 ± 1.3
84 wt. % P(VDF-TrFE)	3.1 ± 0.4
72 wt. % P(VDF-TrFE)	4.2 ± 0.6
56 wt. % P(VDF-TrFE)	3.3 ± 1.0

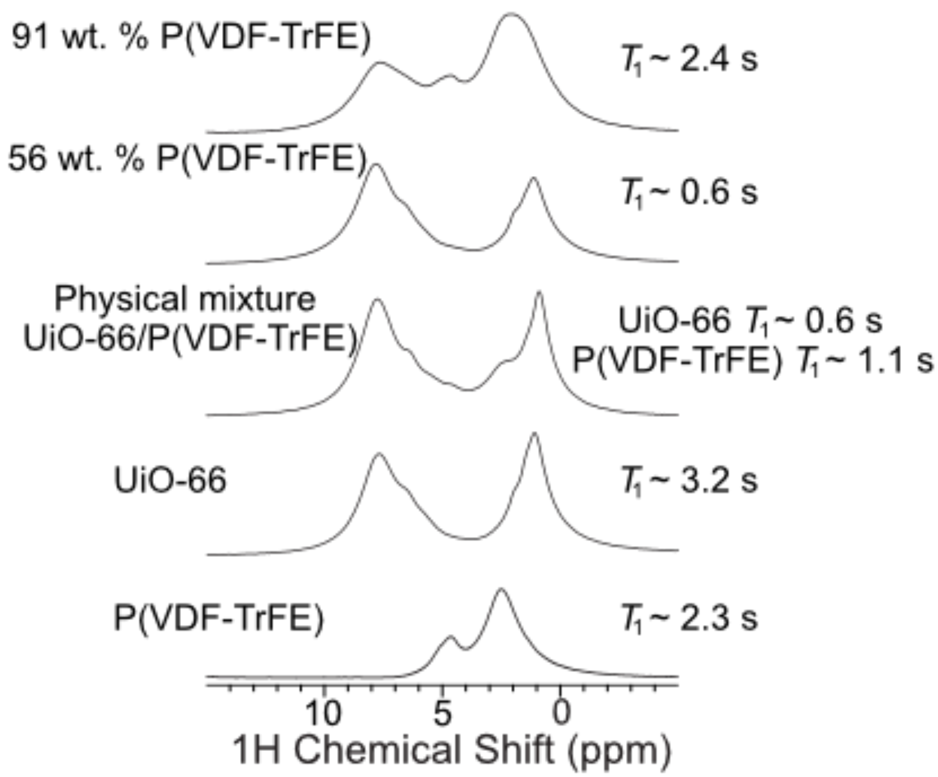

Figure S13: ^{1}H spin echo solid-state NMR spectra obtained with one rotor cycle half-echo delay at 10 kHz MAS frequency and 14.1 T. Indicated ^{1}H longitudinal relaxation times (T_1) were measured using ^{1}H saturation recovery and ^{13}C detection using ^{1}H→^{13}C CP.

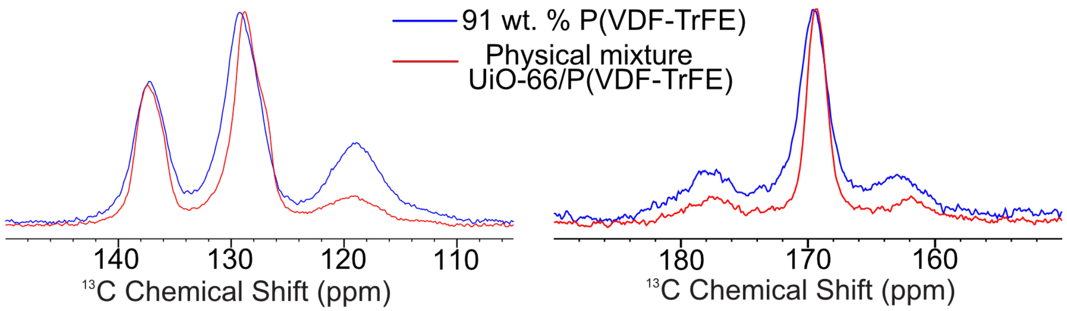

Figure S14: Comparison of ^{1}H→^{13}C CP MAS solid-state NMR spectra of 91 wt. % P(VDF-TrFE) composite and physical mixture of UiO-66 and P(VDF-TrFE).

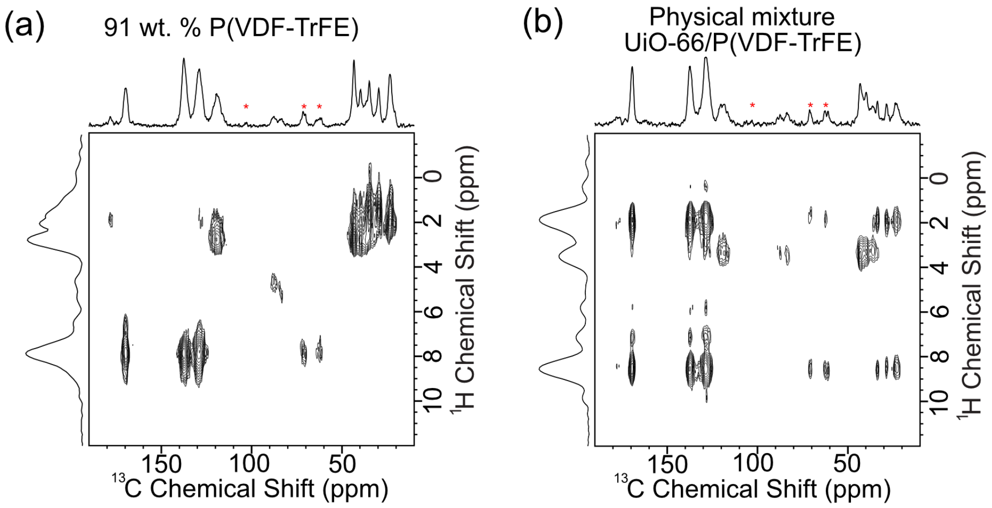

Figure S15: 2D $^{13}C\{^1H\}$ heteronuclear correlation solid-state NMR spectrum of (a) 91 wt. % P(VDF-TrFE) with no spin diffusion, and (b) physical mixture of UiO-66 and P(VDF-TrFE) with 25 ms ^1H-^1H spin diffusion time (t_{sd}). Spinning sidebands are indicated with red asterisks (*).

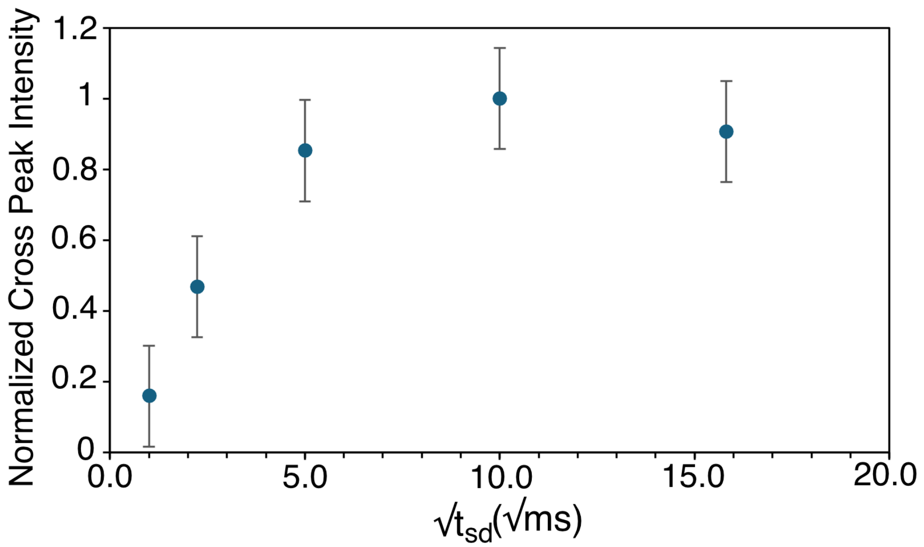

Figure S16: Plot showing intensity of cross peak signals extracted from the 2D spin diffusion $^{13}C\{^1H\}$ heteronuclear correlation solid-state NMR spectra of the 91 wt.% P(VDF-TrFE) composite vs square root of the spin diffusion time (t_{sd}). 2D spectra were obtained at ^1H-^1H spin diffusion periods of 1, 5, 25, 100 and 250 ms. The cross peak intensities were obtained by extracting ^1H slices at a ^{13}C shift of 43 ppm (corresponding to P(VDF-TrFE) signal), and integrating the ^1H signal at the aromatic region corresponding to UiO-66.

Table S3: Table showing sensitivity (V/N) of composite films.

Wt. % of P(VDF-TrFE) in composite films	Sensitivity (V/N)
56	0.171 ± 0.003
72	0.201 ± 0.003
84	0.245 ± 0.005
91	0.262 ± 0.019
100	0.299 ± 0.005

Table S4: Comparison of piezoelectric output voltage and sensitivity reported for MOF–PVDF or MOF–P(VDF-TrFE) composite systems.

	Material System	MOF Type	Fabrication Method	Applied Force	Output Voltage	Sensitivity (V/N)
This work	91 wt.% P(VDF-TrFE)-UiO-66	UiO-66 (Zr-based)	Solution shearing	35 N	~9.1 V	~0.26 V/N
Moghadam et al.[24]	PVDF + UiO-66	UiO-66	Electrospinning	0.536 V	5 N	0.107 V/N
Atighi et al.[39]	PVDF + ZIF-8	ZIF-8	Electrospinning	~2.5 N	~3.84 V	~ 1.536 V/N
You et al.[64]	P(VDF-TrFE) nanofibers	-	Electrospinning	~2kPa	~12 V	-
Tian et al.[63]	P(VDF-TrFE) / nano-Fe_3O_4	-	Electrospinning	12 N	4.6 V	0.38 V/N

In addition to thermal conductivity of the MOF composite, the TDTR measurements are sensitive to variation in the heat capacity of PVDF doped UiO-66 and uncertainties in the transducer thickness, which resulted in large uncertainties in the value. Consequently, no apparent change in thermal conductivity was observed suggesting that any defect scattering from comixing had no effect. These sensitivities are encapsulated in a sensitivity analysis of all parameters in the material stack shown in **Figure S17**. In this analysis, parameters are slightly perturbed to test their influence on the time-variant cylindrical heat equation.

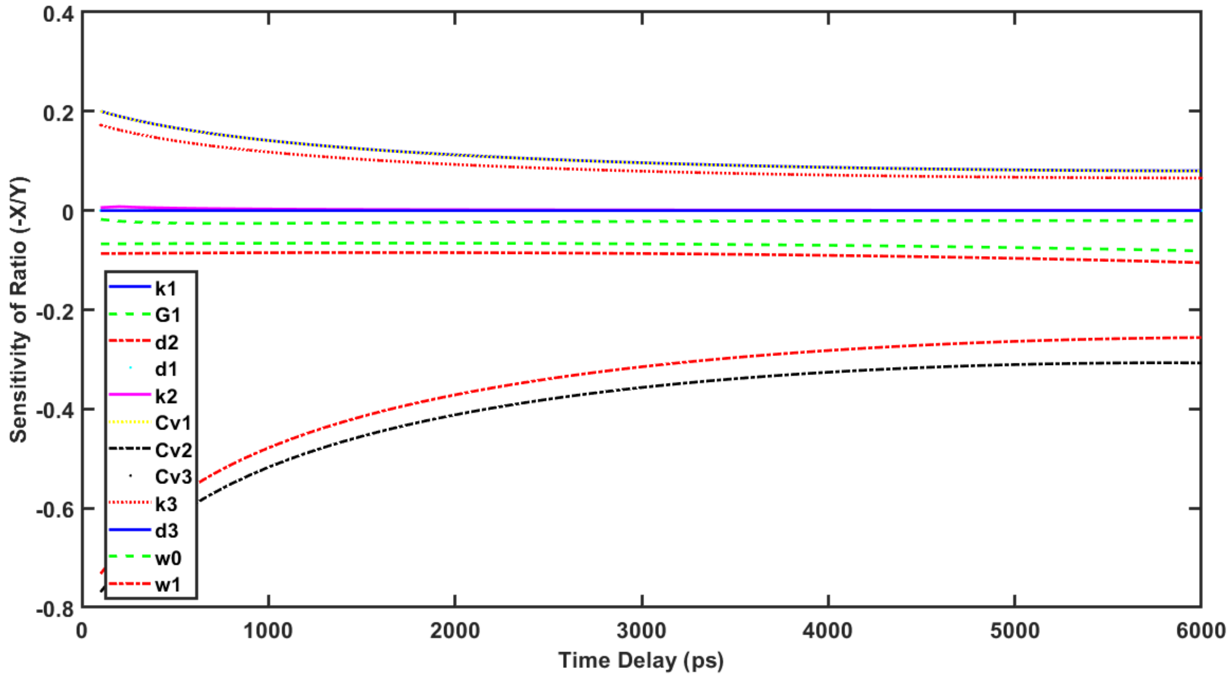

Figure S17: Sensitivity plot showing sample stack parameters and their sensitivity in the cylindrical heat equation. k1,Cv1, d1, G1 are substrate (SiO2) thermal conductivity, heat capacity, thickness, and thermal boundary conductance respectively. k2, Cv2, and d2 are transducer (Al) thermal conductivity, heat capacity, and thickness respectively. k3, Cv3, and d3 are thermal conductivity, heat capacity, and thickness of the measured material. w0 and w1 are spot sizes of the probe and pump beam. Parameters with greater deviation from zero are more measurement-sensitive. We find the largest sensitivity to transducer thickness and material heat capacity, so most uncertainty propagated from these parameters.

Raw data is measured as the ratio between in-phase voltage (heating due to the pump pulse) and out-of-phase voltage (residual heating effects) which improves upon measurement precision. Displayed in **Figure S18** is the raw data of the P(VDF-TrFE) series compared to a bare substrate of Al on SiO2. All data from the P(VDF-TrFE) series overlapped suggesting very similar thermal conductivities.

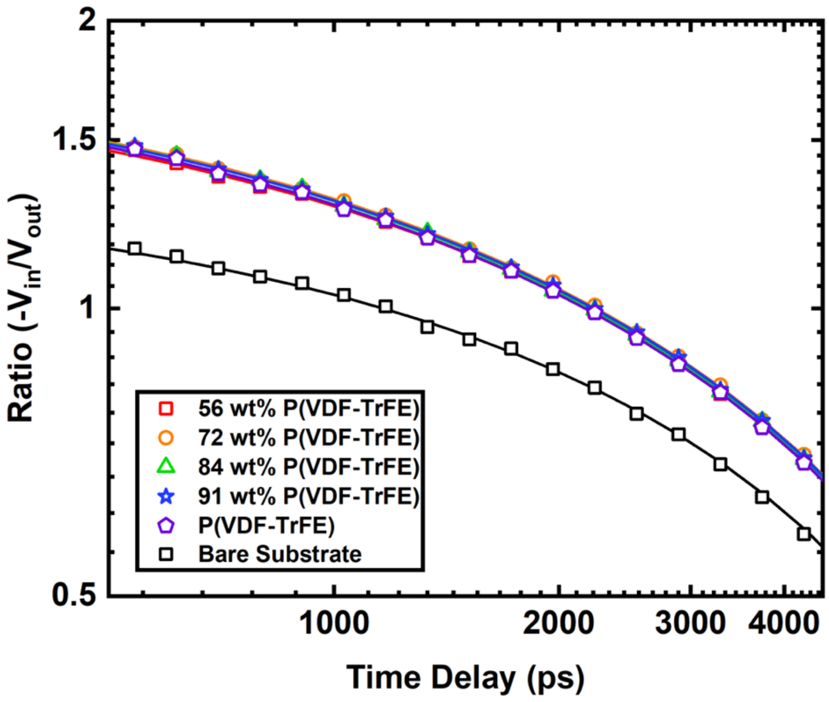

Figure S18: Ratio of in-phase and out-of-phase TDTR data vs time delay for varying weight % P(VDF-TrFE) and UiO-66 co-mixed. Raw data from all the samples overlap well indicating marginal variance in thermal conductivity. A bare substrate of SiO_2 coated with an 80 nm aluminum transducer was included for reference.

Table S5: Anodic and cathodic currents and potentials of the respective modifications derived from CVs in Figure 5d.

**Material**	**E_a (V)**	**I_a (μA)**	**E_c (V)**	**I_c (μA)**	**ΔE (V)**
P(VDF-TrFE)	0.1216	102.8	0.2808	-105.3	-0.1592
72 wt. % P(VDF-TrFE)-UiO-66	0.1163	93.11	0.2838	-78.15	-0.1675
84 wt. % P(VDF-TrFE)-UiO-66	0.1110	97.58	0.2818	-79.60	-0.1708
91 wt. % P(VDF-TrFE)-UiO-66	0.1246	111.8	0.2755	-103.6	-0.1509
